# Effect of seasonal exposure in aeroallergen-sensitised patients with irritable bowel syndrome-diarrhoea

**DOI:** 10.3389/falgy.2025.1568595

**Published:** 2025-05-08

**Authors:** Carlo Maria Rossi, Marco Vincenzo Lenti, Stefania Merli, Martina Fiorita, Antonio Lo Bello, Mario Andrea Latorre, Paola Ilaria Bianchi, Nicola Aronico, Annalisa De Silvestri, Antonio Di Sabatino

**Affiliations:** ^1^ Department of Internal Medicine and Medical Therapeutics, University of Pavia, Pavia, Italy; ^2^ First Department of Internal Medicine, Fondazione IRCCS Policlinico San Matteo, Pavia, Italy; ^3^ Biostatistics and Clinical Trial Centre, Fondazione IRCCS San Matteo, Pavia, Italy

**Keywords:** bowel, irritable bowel syndrome, mast cell, pollinosis, season

## Abstract

**Background:**

Pollen allergy may influence irritable bowel syndrome (IBS) symptoms; however, available data are scant.

**Aims:**

This study aims to assess symptom variability in atopic IBS patients.

**Methods:**

We retrospectively analysed consecutive adult IBS patients evaluated between 2021 and 2024. Patients from the overall IBS cohort and the IBS-diarrhoea (IBS-D) subgroup were classified according to their sensitisation into grass-positive, house dust mite (HDM)-positive, or unsensitised. Symptom burden was assessed using the gastrointestinal symptom rating scale (GSRS) and a visual analogue scale for abdominal pain/distension, both outside the season period (T0) and during the pollination season (T1).

**Results:**

A total of 61 IBS patients were recruited (median age 34 years, IQR 25–50, F:M ratio 3.6:1), including 38 patients (62.8%) with IBS-D (median age 30 years, IQR 28–47, F:M ratio 2.8:1). Atopy was common in the IBS-D subgroup, particularly with respiratory manifestations. The mean GSRS significantly (*p* < 0.01) increased at T1 (variance of 3.4 points) only in grass-sensitised patients as opposed to those sensitised to HDM or unsensitised ones; this effect was present only in the IBS-D subgroup, while no significant variation was observed in the overall cohort.

**Conclusions:**

Pollination season influences symptoms in IBS-D patients sensitised to seasonal allergens.

## 1 Introduction

Irritable bowel syndrome (IBS) is a prevalent, chronic condition associated with impaired quality of life and various comorbidities, including depression, anxiety, asthma, and other atopic diseases (1).

The pathogenesis of IBS is complex, multifactorial, and not yet fully understood; alterations in the gut microbiota, perturbation in mucosal permeability, and low-grade inflammations are implicated as contributing factors (1). The inflammatory infiltrate contains various cell types including eosinophils, T cells, and mast cells (1, 2). The implication of the mast cells in the pathogenesis of IBS is supported by their close proximity to nerve fibres and calls into question the interplay between allergic responses and disorders of gut–brain interaction. Recent studies have focused on the role of mast cells as mediators in IBS pathogenesis (3), and a personalised approach to therapy that takes into account several disease-related and non-disease-related factors has recently been proposed (4).

The role of food allergens seems plausible due to their physical interaction with the gut mucosa. More precisely, alterations in the mucosal barrier, assessed *in vivo* by confocal laser microscopy, have been shown to occur rapidly after antigen exposure to food antigens in some patients with IBS (5, 6). Moreover, some observations in humans and mouse models suggest a possible role of respiratory allergens (7, 8, 9).

Epidemiologic evidence has hypothesised an association between allergic respiratory diseases and IBS. More precisely, patients with atopic disorders, such as seasonal atopic rhinitis and eczema, appear to have a higher likelihood of developing IBS (7).

In a mouse model, allergic airway diseases have been shown to trigger mucosal immune activation in the colon, characterised by elevated tryptase levels, a marker of mast cell degranulation (8). House dust mice allergens have been detected in the gastrointestinal tract, and this finding led some authors to speculate whether they could contribute to intestinal barrier dysfunction (9). Overall, this evidence has led some authors to propose a new group of IBS with allergic manifestations, the so-called atopic IBS (10).

Starting from these premises, we designed a study to assess whether exposure to inhalant allergens affects intestinal symptoms in patients with IBS-diarrhoea (IBS-D) by assessing validated scales at different time points (namely, during or outside the allergen pollination season).

## 2 Materials and methods

### 2.1 Patient population and study design

This was a monocentric, observational, retrospective study conducted at a tertiary referral centre specialising in the diagnosis and management of patients with both gastrointestinal and allergic diseases (Fondazione IRCCS San Matteo, Pavia).

We retrospectively enrolled all consecutive adult (≥18 years) patients with IBS who were referred from the gastroenterology clinic over the last 4 years (2021–2024) to the allergy clinic for evaluation of respiratory symptoms suggestive of allergic rhinitis or with an established diagnosis of allergic rhinitis. Moreover, the IBS cohort included patients with allergic rhinitis who were referred to the gastroenterology unit due to suspected IBS. The timeframe was restricted to 4 years, as the allergy clinic was established in 2021.

The diagnosis of IBS was made by the treating physician according to the latest internationally recognised Rome IV criteria (11). Hence, colonoscopy was not performed in all patients. Patients were classified according to the IBS subtype into IBS-D, IBS constipation (IBS-C), and IBS-mixed (IBS-M). We then focused the analyses on the IBS-D subgroup of patients.

The diagnosis of allergic rhinitis was established according to the latest internationally recognised criteria (12). Patients underwent skin prick testing and/or specific serum IgE testing to whole extracts and molecular allergens to confirm allergic sensitisation; the tests targeted the most common aeroallergens of the region, including seasonal allergens such as temperate grass (*Phleum pratense*), ragweed/mugwort and pellitory and perennial allergens such as house dust mites (HDMs) (*Dermatophagoides pteronissinus* and *Dermatophagoides farinae*), cat, and dog dander. Mono-sensitised patients, either to grasses or HDMs, were followed over 1 year across two grass flowering seasons (March–June and September–October) to assess clinical symptoms of IBS using validated symptom scales at two time points: T0 (outside the pollination season) and T1 (during the pollination season).

Exclusion criteria included current or previous allergen immunotherapy, adherence to allergen-free or low-FODMAP diets among IBS patients, unwillingness to participate, and incomplete data regarding ongoing therapy.

Validated symptom scales included the gastrointestinal symptom rating scale (GSRS)-IBS version, a visual analogue scale (VAS) for abdominal pain/distension, and the Bristol stool scale; the number of stools per day was also recorded.

The GSRS-IBS version is a reliable scale developed for use in adult patients and evaluates different symptoms using a seven-point Likert scale, including abdominal pain, diarrhoea, stool features, and the need to have a bowel movement, among others. The questionnaire has a 7-day recall period (13). In addition, GSRS-IBS version scores were calculated separately for upper (items 1–8) and lower (items 9–15) gastrointestinal symptoms.

Demographic (sex, age) and clinical data of patients were extracted from the electronic hospital records and pseudo-anonymised using a pre-defined spreadsheet. Clinical data included clinical manifestations at onset, atopic comorbidities, history of atopic sensitisation profile, relevant histopathological and general laboratory findings, referral details, pharmacologic therapies, and dietary habits. Any missing information not present in the electronic records or physicians’ assessment forms was retrieved through a phone call with the patients. Informed consent was obtained from all participants. The study was performed as a clinical audit using routinely collected clinical and laboratory data. All participants provided written informed consent for the use of their data in an aggregated and anonymised format. The study was approved by the local ethics committee (0023744/22). All results are reported according to the STrengthening the Reporting of OBservational studies in Epidemiology (STROBE) recommendations for quality assurance. In compliance with privacy regulations, raw data cannot be made public but can be shared by the corresponding author upon reasonable request.

### 2.2 Statistical analysis

Continuous data are presented as the median and interquartile range (IQR; i.e., 25th–75th percentiles) or the mean and standard deviation (SD), while categorical data are presented as counts and percentages. Comparisons between the two groups were performed using Student’s *t*-test for continuous variables and Fisher’s exact test for categorical variables. Missing data were excluded from percentage calculations when specified.

Software Stata 18.5 (StataCorp, College Station, TX, USA) was used for all computations. A two-sided *p*-value < 0.05 was considered statistically significant.

## 3 Results

### 3.1 Demographic, clinical, and laboratory data

A total of 61 IBS patients (median age 34 years, IQR 25–50, F:M ratio 3.6/1) were recruited. Of these, 38 (62.2%) were classified as IBS-D (median age 30 years, IQR 28.2–47.7, F:M ratio 2.8:1), 18 (29.5%) as IBS-M (median age 32.5 years, IQR 23.5–46.7, F:M 10:1 ratio), and 5 (8.2%) as IBS-C (median age years 35.0, IQR 31–55, F:M 1.5:1 ratio).

Demographic, clinical, and laboratory features of the overall IBS cohort and the IBS-D subgroup are reported in Supplementary Table S1. As expected, female sex and younger age were common features. However, age and sex did not differ across groups. Smoking and alcohol consumption were frequent in both patient groups. Atopy was also a common finding in both groups, with respiratory manifestations (i.e., allergic rhinitis and asthma) being the most frequent, particularly allergic rhinitis. Eczema and nasal polyposis were not observed in the IBS-D subgroup of patients. Of note, serum IgE levels and eosinophil counts were similar.

In the overall cohort of IBS patients, the initial referral was equally distributed between the allergology and gastroenterology clinics (*n* = 30/61 and *n* = 31/61, respectively; data not shown in Table 1), whereas in the IBS-D subgroup of patients, the initial referral was allergological (*n* = 22/38, and 16/38, respectively; data not shown in Supplementary Table S1).

**Table 1:** Comparison of the mean scores of clinical symptom scales at the two different time points within groups defined according to the sensitisation profile to grasses or house dust mites in the overall IBS patient cohort.

Parameter	Grass-allergic patients			House dust mite-allergic patients			Unsensitised patients		
	T0	T1	*p*-value	T0	T1	*p*-value	T0	T1	*p*-value
GSRS score	24.3 ± 6.6	26.29 ± 8.2	0.17	21.2 ± 8.2	20.1 ± 7.4	0.37	21.2 ± 8.2	20.1 ± 8.2	0.14
U-GSRS score	8.5 ± 1.1	8.5 ± 1.0	>0.99	9.4 ± 2.1	9.7 ± 2.8	0.33	8.9 ± 1.9	8.8 ± 1.7	0.70
L-GSRS score	16.1 ± 5.7	18.5 ± 7.4	0.07	15.3 ± 7.3	16.8 ± 7.6	0.28	12.1 ± 4.8	12.0 ± 4.8	0.12
Abdominal pain distension (VAS)	3.1 ± 3.3	3.4 ± 3.5	0.78	3.1 ± 2.3	3.7 ± 2.4	0.15	3.1 ± 2.3	2.7 ± 2.4	0.12

GSRS, gastrointestinal symptoms rating scale; L, lower gastrointestinal tract; HDM, house dust mite; U, upper gastrointestinal tract; VAS, visual analogue scale.

Anti-allergic and other therapies for the two groups are reported in Supplementary Table S2. Only three patients in the overall IBS cohort, including two in the IBS-D subgroup, were receiving anti-diarrhoeal agents.

Nearly 20% of patients in the two groups were receiving topical nasal therapy (nasal steroids with or without topical antihistamines or nasal vasoconstrictor), administered continuously for at least 2 weeks. Only four patients in the overall IBS cohort, including two in the IBS-D subgroup, received oral antihistamines.

The median GSRS and VAS scores for abdominal distension did not significantly differ between the overall IBS group and the IBS-D subgroup; median GSRS scores were 22.5 (IQR 18.0–29.0) and 25.5 (IQR 18.0–29.7), respectively (*p* = 0.5), and median VAS scores were 2.0 (IQR 1.0–5.0) and 2.5 (IQR 1.0–5.0), respectively (*p* = 0.9).

### 3.2 Sensitisation profile of patients with IBS and its variants

Supplementary Figure S1 shows the sensitisation profiles of the overall cohort of patients with IBS, the IBS-D subgroup, and the other variants—IBS-M and IBS-C pooled together—according to the sensitisation to a seasonal respiratory allergen (grass) or a perennial one (HDM). Nearly 50% of IBS and IBS-D patients were sensitised to either grass or HDM, accounting for 28 and 20 sensitised patients, respectively. The low rate of allergic sensitisation among patients with other IBS variants prevented further analysis, as presented in Supplementary Table S1.

All principal classes of pan-allergens relevant to the region were observed among patients in the IBS-D subgroup, as presented in Supplementary Table S1.

### 3.3 Variability of IBS symptom scales according to the sensitisation profile in the overall cohort of patients with IBS and the IBS-D subgroup

To assess whether IBS symptoms varied according to respiratory allergen exposure in our cohort of IBS patients, specifically in the IBS-D subgroup, we compared standardised scales evaluating gastrointestinal symptoms using the GSRS, including its subscales, and the VAS for abdominal pain/distension across two different time points; these comparisons were made among patients sensitised to grass, those sensitised to HDM (a perennial allergen with presumably stable house concentration, serving as a positive control), and unsensitised patients (serving as a negative control), as presented in Tables 1 and 2.

**Table 2:** Comparison of the mean scores of clinical symptom scales at the two different time points within groups defined according to the sensitisation profile to grasses or house dust mites in the IBS-D subgroup.

Parameter	Grass-allergic patients			House dust mite-allergic patients			Unsensitised patients		
	T0	T1	*p*-value	T0	T1	*p*-value	T0	T1	*p*-value
GSRS score	25.3 ± 7.4	28.7 ± 8.2	**<0**.**01**	24.3 ± 6.2	24.6 ± 6.6	0.4	22.4 ± 7.4	22.0 ± 7.4	0.4
U-GSRS score	8.5 ± 1.0	8.5 ± 1.3	>0.9	9.6 ± 2.2	9.8 ± 3.1	>0.9	8.9 ± 2.2	8.8 ± 3.1	0.5
L-GSRS score	15.9 ± 5.9	18.8 ± 7.1	**0**.**01**	16.4 ± 8.2	17.2 ± 9.1	0.5	13.5 ± 4.8	12.7 ± 4.9	0.1
Abdominal pain distension (VAS)	2.6 ± 3.0	3.9 ± 3.5	**0**.**03**	3.1 ± 2.8	3.7 ± 3.0	0.06	3.2 ± 2.5	2.7 ± 2.5	0.2

GSRS, gastrointestinal symptoms rating scale; L, lower gastrointestinal tract; HDM, house dust mite; U, upper gastrointestinal tract; VAS, visual analogue scale.

When analysing the GSRS and VAS scores for abdominal pain/distension in the overall IBS cohort of patients (Table 1), no statistically significant variation was found between values during the pollination season (T1) and the basal value outside the pollination season (T0), according to the sensitisation profile; however, a trend towards increased GSRS scores (from 16.1 ± 5.7 to 18.5 ± 7.4) was observed in patients sensitised to grass, specifically in the GSRS component dealing with lower gastrointestinal tract symptoms.

In the IBS-D subgroup (Table 2), the mean GSRS score significantly increased (*p* = 0.04) in grass-allergic patients, from 25.3 ± 7.4 (T0) to 28.7 ± 8.2 (T1), while no statistically significant variation was observed in patients sensitised to HDM or in unsensitised patients (*p* = 0.4 and *p* = 0.4, respectively). Of note, this increase in GSRS was related to the lower gastrointestinal tract component, which significantly increased across time points from 15.9 ± 5.9 to 18.8 ± 7.1 (*p* = 0.01), while the component reflecting upper gastrointestinal symptoms remained unvaried (8.5 ± 1.0 and 8.5 ± 1.3, *p* > 0.9).

Moreover, only in the IBS-D subgroup of patients and specifically only in those sensitised to grass, the mean VAS score for abdominal pain/distention also significantly increased (*p* = 0.03), from 2.6 ± 3.0 (T0) to 3.9 ± 3.5 (T1), compared to those sensitised to HDM (*p* = 0.06) or unsensitised patients (*p* = 0.2), as presented in Table 2.

**Figure 1:**
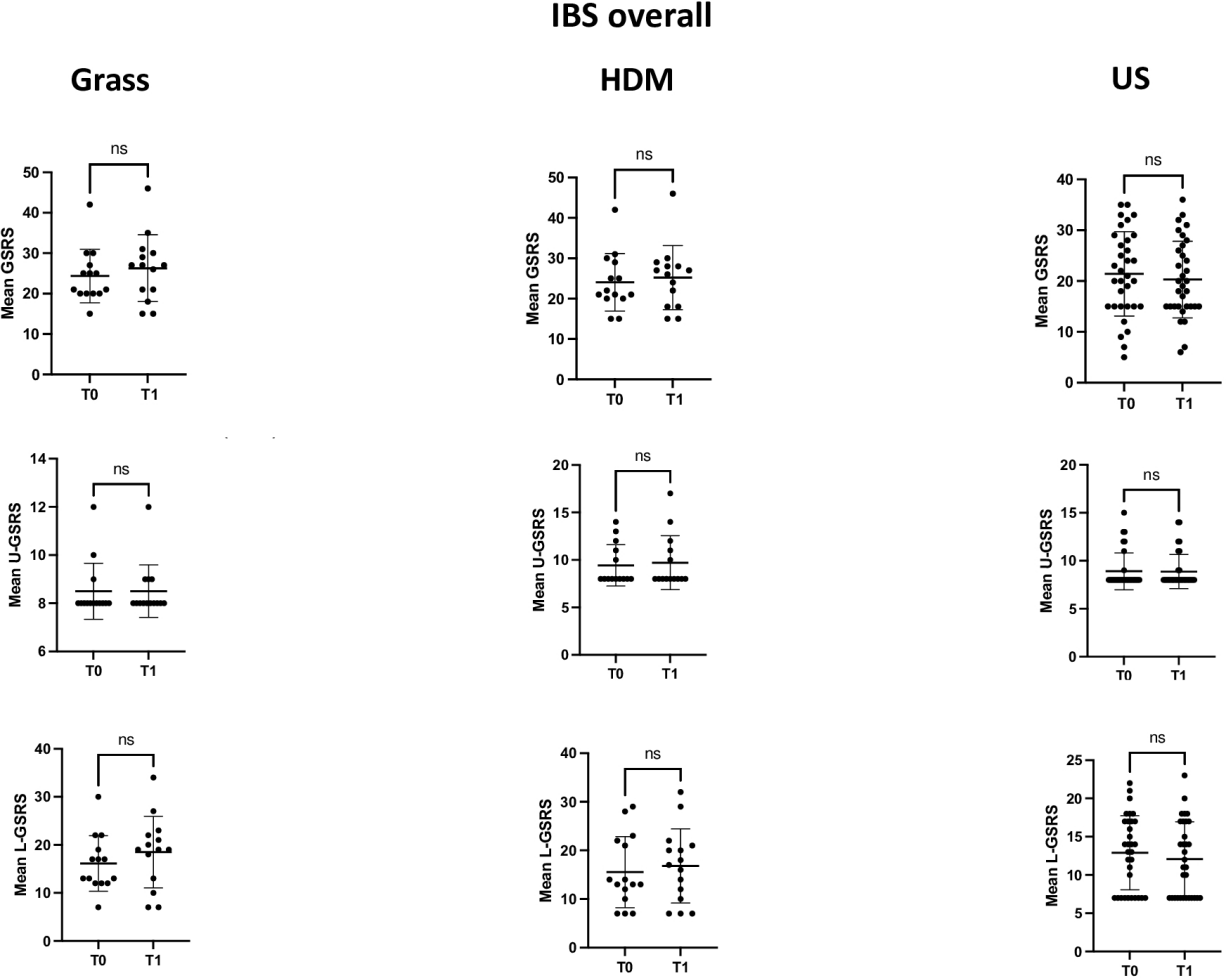
Comparison of the mean GSRS and its components dealing with upper (U-GSRS) or lower (L-GSRS) gastrointestinal tract symptoms at the two time points (T0 and T1) in the overall IBS patient cohort, according to the sensitisation profile to grasses and HDMs. For grass-allergic patients, T0 denotes the outside the season, while T1 denotes the pollination season.

**Figure 2:**
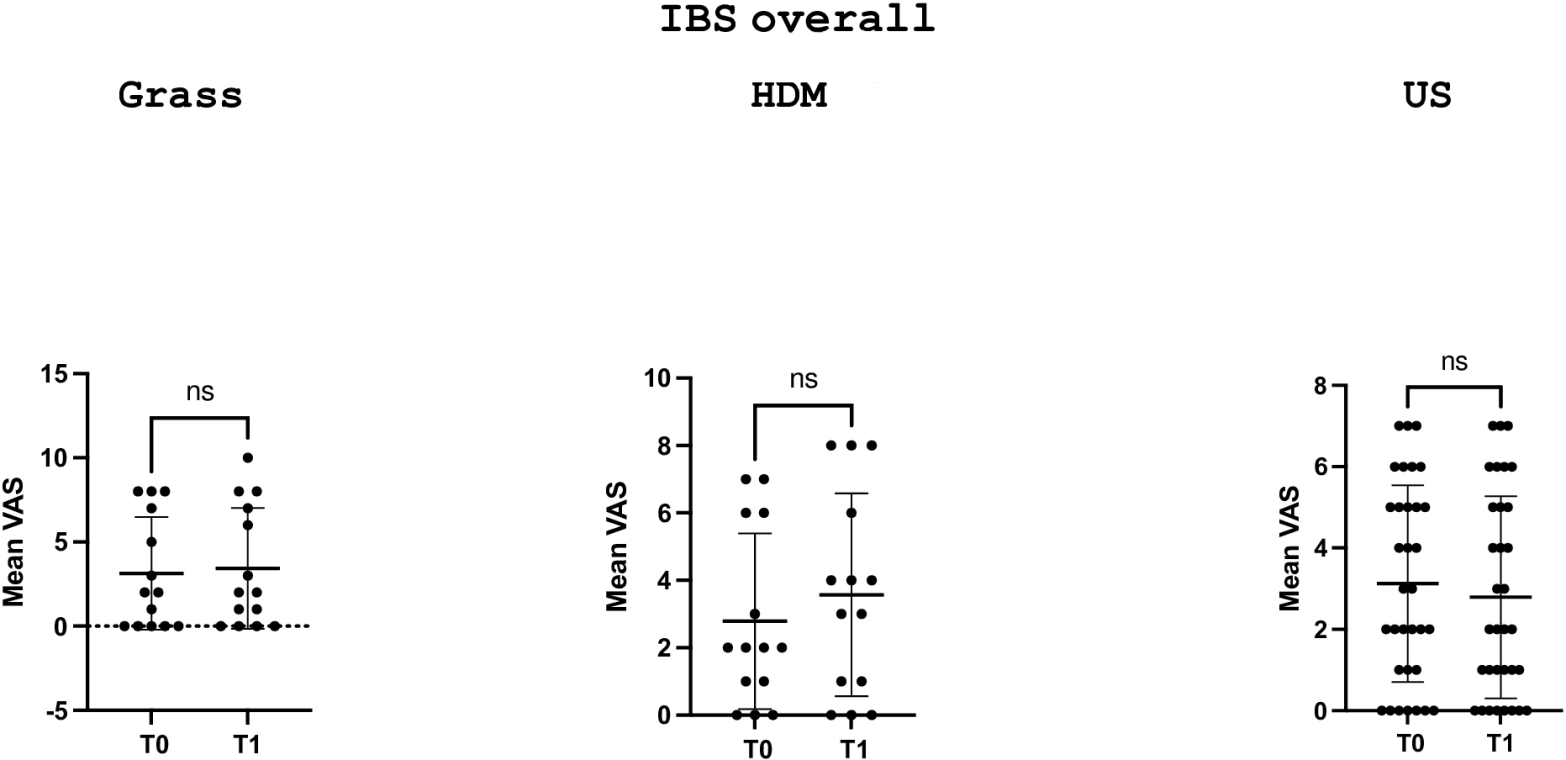
Comparison of the VAS scores for abdominal distension/pain at the two time points (T0 and T1) in the overall IBS cohort according to the sensitisation profile to grasses and HDMs. US denotes unsensitised patients. For grass-allergic patients, T0 denotes the outside the season, while T1 denotes the pollination season.

**Figure 3:**
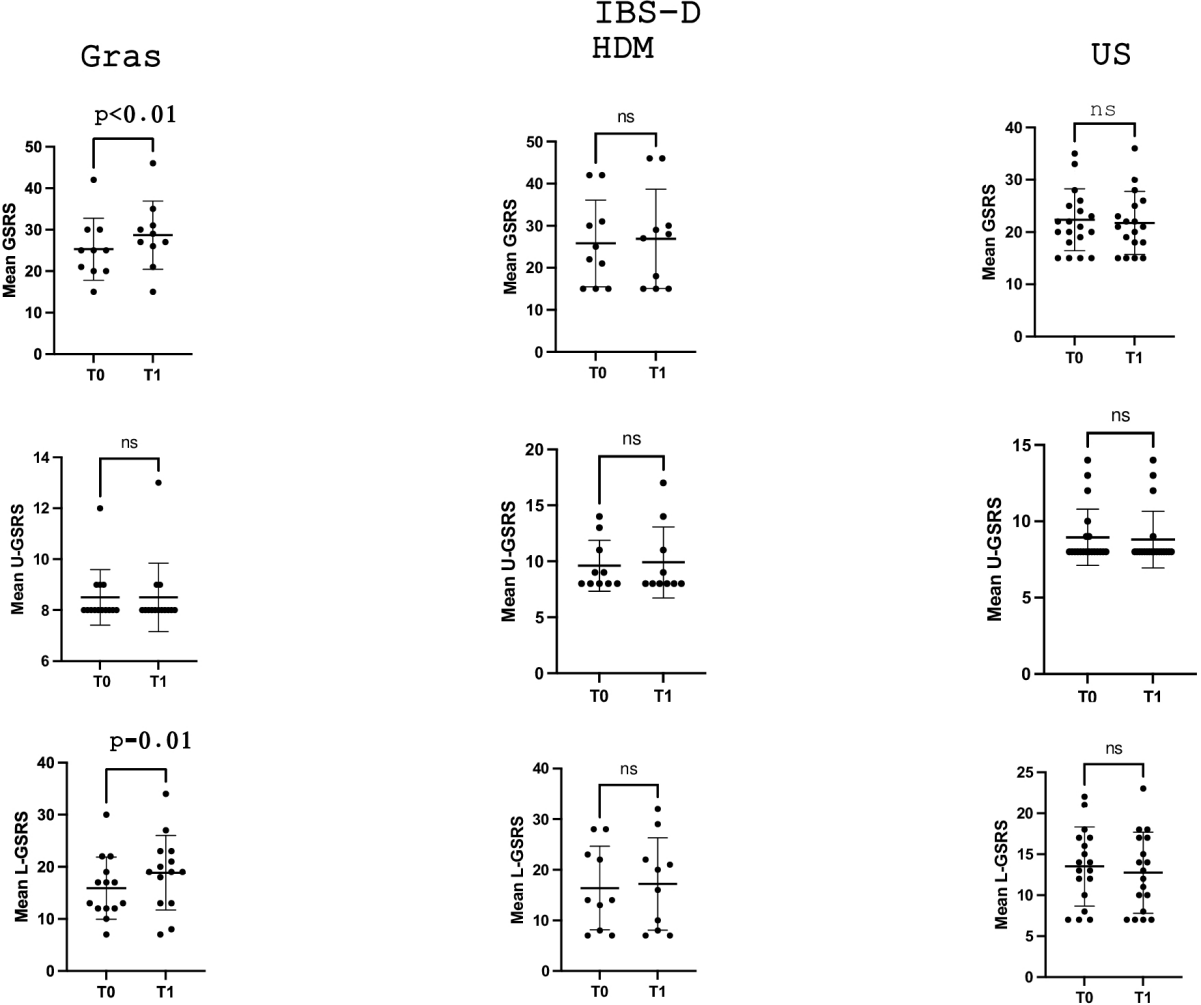
Comparison of the mean GSRS and its components dealing with U-GSRS or L-GSRS gastrointestinal tract symptoms at the two time points (T0 and T1) in the IBS-D subgroup according to the sensitisation profile to grasses and HDMs. For grass-allergic patients, T0 denotes the outside the season, while T1 denotes the pollination season.

**Figure 4:**
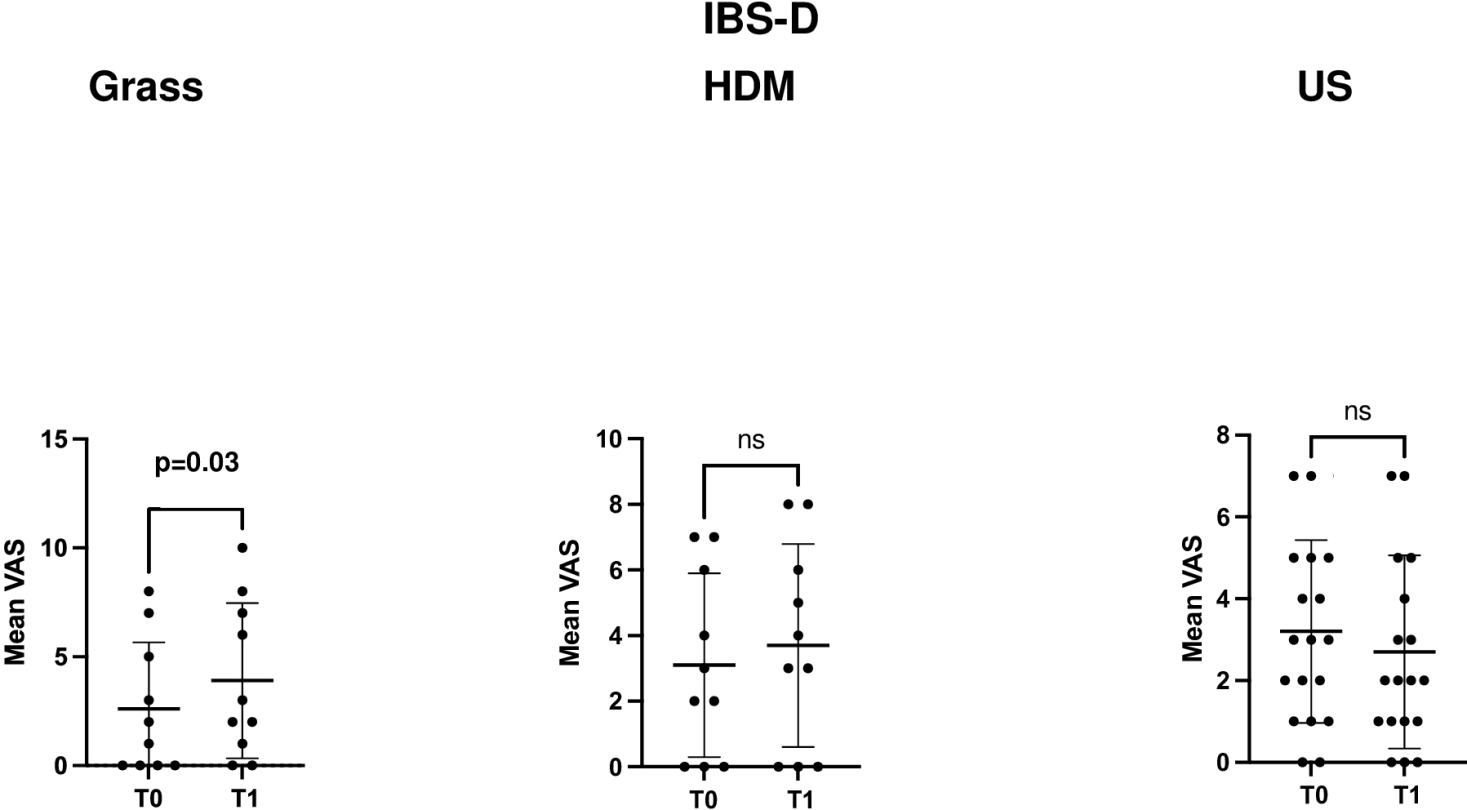
Comparison of the VAS scores for abdominal distension/pain at the two time points (T0 and T1) in the IBS-D patient subgroup according to the sensitisation profile to grasses and HDMs. US denotes unsensitised patients. For grass-allergic patients, T0 denotes the outside the season, while T1 denotes the pollination season.

Figures 1 and 3 depict the variation in the GSRS score and its components across time points in the three groups, defined according to the sensitisation profile in the overall cohort patients with IBS and the IBS-D subgroup, respectively, while Figures 2 and 4 depict the variation in VAS scores for abdominal pain/distension across time points in the three groups, defined according to the sensitisation profile in the overall cohort of patients with IBS and the IBS-D subgroup, respectively.

The magnitude of variation in the GSRS, its components, and the VAS score for abdominal pain/distension across the different groups is also presented in Supplementary Table S3.

### 3.4 Factors associated with IBS symptoms variability in bivariate analysis in the subgroup of patients with IBS-D

Given the observed variability in IBS symptoms, as assessed by the GSRS, in the subgroup of patients with IBS-D, we looked into associated factors using a bivariate regression model, including sex, age at diagnosis, history of atopy, allergic comorbidities (including atopic dermatitis), total IgE levels, and specific IgE levels for grass as covariates. Among these covariates, only male sex was found to be statistically significant (1.69 coefficient, 95% CI 0.04–3.34, *p* = 0.04).

## 4 Discussion

The pathogenesis of IBS is complex, multifactorial, and remains partly elusive. Among known factors, alterations in gut motility, visceral hypersensitivity, dysbiosis of the gut microbiota, disturbance in gut–brain interactions, bacterial overgrowth, prior infections, and immune activation characterised by low-grade inflammation have a role (14).

More recently, alterations in the gut barrier function and altered permeability have been recognised as key contributors (15). In this context, mast cells have received much attention due to their localisation in proximity to mucosal surfaces and vascular beds, the vasoactive effects of their granular contents (including histamine, among others), and their interaction with nerve fibres (16). Moreover, mast cell mediators are known to influence gut motility and secretion.

The cause of symptom variation in IBS remains currently unexplained. Yet, it is possible that environmental factors, such as allergen exposures, may play a role.

Of note, in eosinophilic esophagitis, a chronic immune-mediated condition of the oesophagus characterised by type 2 inflammation and frequent allergic sensitisation, the effectiveness of allergen-free diets appears reduced during the pollination season (17). More precisely, pollen-sensitised patients displayed a significantly lower response to the six-food elimination diet during the pollination season compared with outside the pollination season (21.4% vs. 77.3%; *p* = 0.003); their response was also lower compared to unsensitised patients (21.4% vs. 77.8%; *p* = 0.01).

Under this point of view, we therefore decided to evaluate whether symptom burden in a cohort of adult patients with IBS is influenced by allergenic exposures. The IBS-D subgroup was chosen because it was quantitatively larger and, above all, because mast cells seem to be more implicated in the pathogenesis of diarrhoea through the release of active mediators altering gut motility rather than constipation. Moreover, the frequency of allergic sensitisation was higher in the IBS-D subgroup than in the other subgroups.

In this monocentric work, we showed that IBS patients present frequent atopic comorbidities, similar to other immune-mediated disorders of the lower gastrointestinal tract (18, 19). Moreover, our data incorporating different aspects of IBS symptom burden, as assessed by the GSRS and VAS for abdominal distention/pain, revealed that pollen exposure significantly impacts only the subgroup of patients with IBS-D sensitised to seasonal respiratory allergens (grass), with symptoms worsening during the pollination season, although a similar trend was also observed in the overall cohort of patients with IBS. The symptom burden was related to lower gastrointestinal tract symptoms, as assessed by the items of the GSRS dealing with colonic symptoms. No such variation was found in patients sensitised to HDM. It is possible that an increase in symptoms during winter months was not observed in this subset of patients due to high adherence to environmental interventions for HDM control, such as regular vacuuming and the use of pillow/mattress encasements, among others. Indeed, these patients were managed in a third-level centre, where the importance of preventive measures is usually stressed, especially for patients not undergoing immunotherapy. Unfortunately, data on interventions for HDM control were not collected.

The mechanistic interpretation of our findings remains elusive. It is possible that during the pollination season, a large amount of respiratory allergens is partly swallowed, and aeroallergens may have a direct effect on colon mast cells, enhancing their degranulation rate, or an indirect effect through cross-reactivity with food allergens. Although pollen allergens are generally considered acid-labile, protein pump inhibitor use and hypochlorhydria may leave these allergens intact in more distal segments of the gut. Moreover, greater duodenal infiltration of eosinophils and mast cells has been observed in biopsies taken during birch pollen season compared to biopsies taken outside the seasons in patients with birch pollinosis and birch-plant syndrome (20, 21).

Alternatively, respiratory airway inflammation may influence gut mast cell activity through circulating mediators, such as alarmins, like IL25, IL33, and thymic stromal lymphopoietin (TSLP).

Finally, we found male sex as a factor increasing the variability of intestinal symptoms.

Different and possibly not mutually exclusive explanations may be put forward to account for this finding. One possibility is that men are more influenced by environmental pollen exposure due to increased outdoor activities compared to women. This finding could also underpin a biological phenomenon related to hormonal factors. However, some clinical and experimental observations are at odds with this finding since oestrogens were shown to upregulate genes relevant to mast cells in a mouse model of endometriosis (22). Moreover, females with allergic rhinitis were also found to exhibit a more pronounced neurogenic response, possibly mediated by mast cells, after nasal challenge (23). As a consequence, larger studies are needed to ascertain the role of sex as a cofactor for symptom variability in this subset of IBS patients.

Among the strengths of our study, to be noted are the in-depth allergy characterisation of patients, the low prevalence of systemic medications, including antihistamines, which may perturb mast cell functions also at the gut level and may represent a confounding factor for assessing symptom burden, and the presence of two highly selected control groups, represented by patients accounting for the allergy sensitisation profile.

Our preliminary study has some limitations, including the small sample size, its monocentric design, the potential for selection bias due to patient recruitment from an allergy clinic, and the possible recall bias for GSRS due to the retrospective collection of GSRS data. Also, our data can only be generalised to a similar setting; therefore, larger studies are needed to validate our results and should also consider including polysensitised patients and patients with other IBS subtypes. Yet, the finding that symptom burden in IBS may be influenced by pollen exposure could have some implications for patient monitoring and clinical management, especially during the pollination season, where anti-diarrhoeal treatment may need intensification. Moreover, it provides a rationale for setting up studies aimed at testing whether mast cell-targeted therapies, such as antihistamines and chromones, among others, may exert beneficial effects on IBS management.

## Data Availability

The raw data supporting the conclusions of this article will be made available by the authors upon reasonable request.

## Supplementary material

Supplementary Figure S1 Flowchart showing the groups of patients characterised by the sensitisation profile into grass-positive, house dust mite (HDM)-positive, and unsensitised (US) in the overall IBS cohort, IBS-D subgroup, and IBS-C/IBS-M subgroups pooled together.
