# Supplementary material for: Effect of seasonal exposure in aeroallergen-sensitised patients with irritable bowel syndrome-diarrhoea
Source: Front Allergy. 2025 May 8;6:1568595. doi: 10.3389/falgy.2025.1568595 (PMC12095291; doi:10.3389/falgy.2025.1568595)
Supplement: Supplementary Figure S1 — Flowchart showing the groups of patients characterised by the sensitisation profile into grass-positive, house dust mite (HDM)-positive, and unsensitised (US) in the overall IBS cohort, IBS-D subgroup, and IBS-C/IBS-M subgroups pooled together. [file Table1.docx]

**Supplementary Table 1**. Main sociodemographic and clinical features of the overall irritable bowel syndrome (IBS) patient cohort, including those with IBS- diarrhoea subtype (IBS-D)

| **Parameter*** | **Overall IBS (N=61)** | **IBS-D subgroup (N=38)** |
| --- | --- | --- |
| Demographical factors | | |
| Age (years) | 34 (26.0-50.0) | 30 (28.2-47.7) |
| Sex female (%) | 48 (78.6) | 28 (73.7) |
| Alcohol consumption (%) | 15 (24.5) | 9 (23.7) |
| Smoking (%) | 12 (19.7) | 9 (23.7) |
| Clinical manifestations | | |
| Constipation (%) | 5 (8.2) | / |
| Diarrhoea (%) | 38 (50.8) | 38 (50.8) |
| Mixed (%) | 18 (29.5) | / |
| Abdominal distension (%) | 48 (78.7) | / |
| Atopy (%) | 26 (42.6) | 18 (47.3) |
| Laboratory parameters | | |
| Faecal calprotectin (microg/g) | 30 (20-40) | 26 (20-33) |
| Total IgE (kU/l) | 119 (34-363) | 106 (37-597) |
| Serum eosinophils (n°/mm3) | 130 (100-220) | 120 (92-190) |
| Atopic comorbidities | | |
| Eczema (%) | 2 (3.3) | 0 (0) |
| Allergic rhinitis (%) | 24 (39.6) | 16 (42.1) |
| Asthma (%) | 7 (11.4) | 4 (10.5) |
| Nasal polyposis (%) | 1 (2.5) | 0 (0) |
| Food allergy (%) | 5 (8.2) | 1 (2.6) |
| Allergic sensitization profile^!^ | | |
| House dust mites | 15/61 | 10/38 |
| Grasses | 14/61 | 10/38 |
| Unsensitized | 32/61 | 20/38 |
| Molecular allergens | | |
| PR-10 | 4/61 | 1/38 |
| Profilin | 3/61 | 1/38 |
| LTP | 1/61 | 1/38 |

*Parameters are expressed as median and interquartile range (continuous variables) or proportions (categorical variables).

^!^Either skin prick test or specific IgE (>0.1 K/uL) positivity. In patients with IBS, the percentage of sensitizations was calculated only in those with diarrhea (IBS-D)

Abbreviations. LTP, lipid transfer protein; PR, pathogenesis-related.

**Supplementary Table 2**. General and anti-allergic therapies in the overall cohort of patients with irritable bowel syndrome (IBS), including IBS-diarrhoea subtype (IBS-D)

| **Parameters** | **Overall IBS (N=61)** | **IBS-D subgroup(N=38)** |
| --- | --- | --- |
| GFD (%) | 0 (0.0) | 0 (0.0) |
| Low FODMAP diet (%) | 1 (1.6) | 1 (2.6) |
| Antidiarrheal agents (%)^!^ | 3 (4.9) | 2 (5.2) |
| Lactose avoidance | 2 (3.2) | 1 (2.6) |
| Nasal topical steroid+ topical antihistamines (%) | 5 (8.1) | 3 (7.8) |
| Nasal Topical steroid (%) | 5 (8.1) | 4 (10.5) |
| Nasal vasoconstrictor (%) | 2 (3.2) | 1 (2.6) |
| Systemic antihistamines (%)^#^ | 4 (6.5) | 3 (7.8) |

Abbreviations. FODMAP, fermentable oligosaccharides,disaccahrides, monosaccahrides, polyols; GFD, gluten-free diet ^#^for a duration of at least 2 weeks

^!^ for at least 2 days a week

**Supplementary Table 3.** Score change variation of the clinical symptom scales in the IBS-D subgroup of patients, according to the sensitization profile to Grass or house dust mites.

| **Parameter** | **Grass allergic patients** | **House dust mite allergic patients** | **Unsensitised patients** |
| --- | --- | --- | --- |
| GSRS score | 3.4 | 0.3 | -0.4 |
| U-GSRS score | 0.0 | 0.2 | -0.1 |
| L-GSRS score | 2.9 | 0.8 | -0.8 |
| Abdominal pain/distension (VAS) | 1.3 | 0.6 | -0.5 |

Abbreviations. GSRS, gastrointestinal symptoms rating scale, L, lower gastrointestinal tract; HDM, house dust mites; US, unsensitized patients; U, upper gastrointestinal tract, VAS, visual analogue scale.
