# Supplementary material for: Effect of seasonal exposure in aeroallergen-sensitised patients with irritable bowel syndrome-diarrhoea
Source: Front Allergy. 2025 May 8;6:1568595. doi: 10.3389/falgy.2025.1568595 (PMC12095291; doi:10.3389/falgy.2025.1568595)
Supplement: Supplementary file 2 [file Presentation1.pptx]

## Slide 1
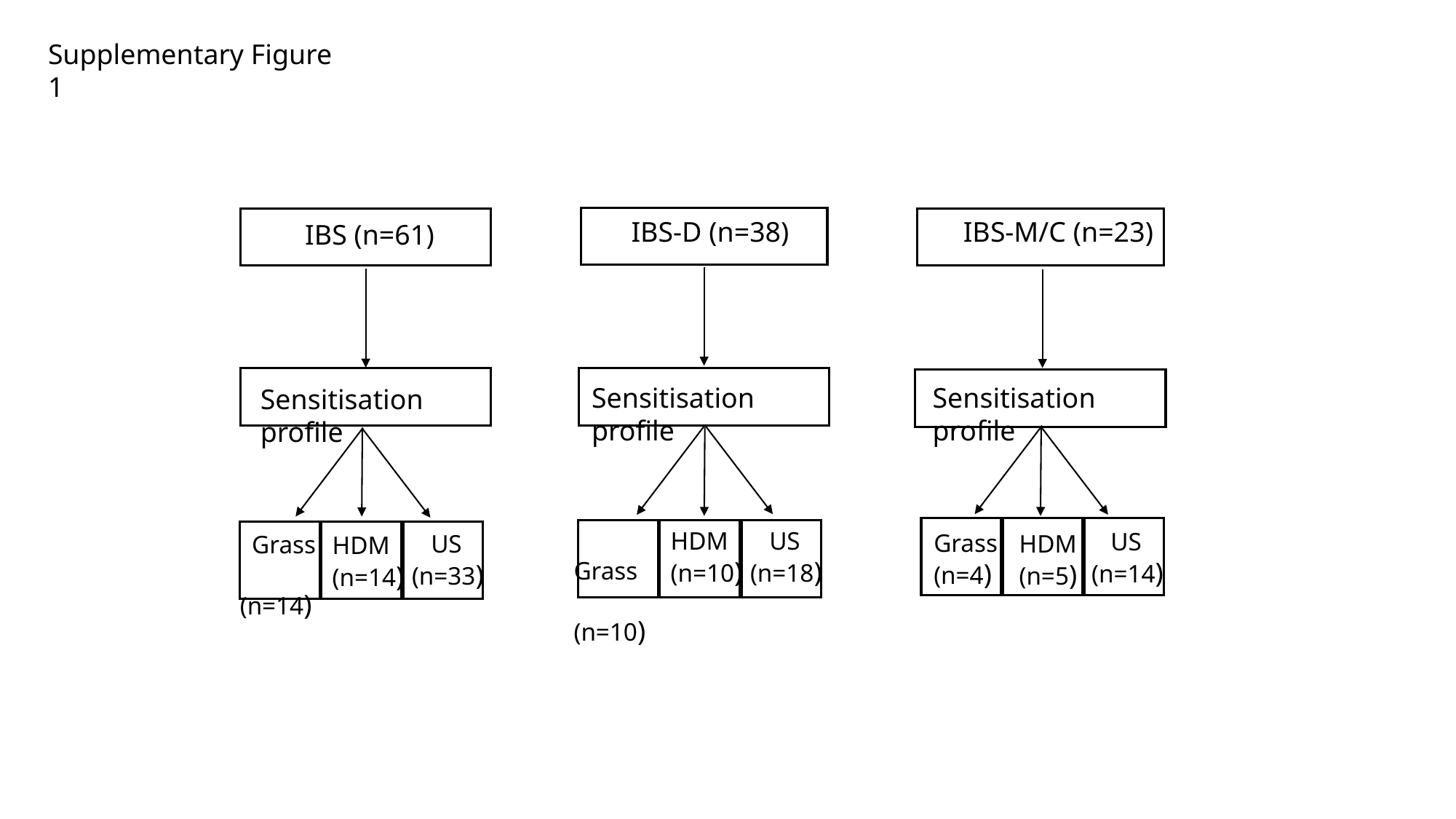

Supplementary Figure 1
IBS-D (n=38)
 IBS-M/C (n=23)
IBS (n=61)
Sensitisation profile
Sensitisation profile
Sensitisation profile
HDM
(n=10)
 US
(n=18)
 Grass
 (n=10)
 US
(n=14)
 Grass
 (n=4)
HDM
(n=5)
 US
(n=33)
 Grass
 (n=14)
HDM
(n=14)
